# Long non-coding RNA KCNQ10T1/miR-19a-3p/SMAD5 axis promotes osteogenic differentiation of mouse bone mesenchymal stem cells

**DOI:** 10.1186/s13018-023-04425-w

**Published:** 2023-12-06

**Authors:** He Lin, Lanjun Nie, Guiqing Lu, Haixia Wu, Tao Xu

**Affiliations:** 1 Department of Plastic Surgery, BenQ Medical Center, The Affiliated BenQ Hospital of Nanjing Medical University, No.71, Hexi Street, Jianye District, Nanjing, 210019 Jiangsu Province China; 2 Dermatological Department, BenQ Medical Center, The Affiliated BenQ Hospital of Nanjing Medical University, Nanjing, Jiangsu Province China; 3 Department of Neurosurgery, BenQ Medical Center, The Affiliated BenQ Hospital of Nanjing Medical University, Nanjing, Jiangsu Province China

**Keywords:** Bone mesenchymal stem cells, KCNQ10T1, Osteogenic differentiation, miR-19a-3p, SMAD5

## Abstract

**Background:**

Bone fracture is a common orthopedic disease that needs over 3 months to recover. Promoting the osteogenic differentiation of bone mesenchymal stem cells (BMSCs) is beneficial for fracture healing. Therefore, this research aimed to study the roles of long non-coding RNA (lncRNA) KCNQ10T1 in osteogenic differentiation of BMSCs.

**Methods:**

BMSCs were treated with osteogenic medium and assessed by CCK-8 and flow cytometry assays. Alkaline phosphatase (ALP) staining, alizarin red staining (ARS), as well as concentration of osteoblast markers were measured to evaluate osteogenic differentiation of BMSCs. Western blot was employed to detect proteins; while, qRT-PCR was for mRNA levels. Additionally, targeted relationships between KCNQ10T1 and miR-19a-3p, as well as miR-19a-3p and SMAD5 were verified by dual luciferase reporter gene assay along with RNA pull-down method.

**Results:**

Upregulation of KCNQ10T1 promoted the ALP staining and ARS intensity, increased the cell viability and decreased the apoptosis rate of BMSCs. Besides, KCNQ10T1 overexpression increased the ALP, OPG, OCN and OPN protein levels. KCNQ10T1 sponges miR-19a-3p, which targets Smad5. Upregulated miR-19a-3p reversed the overexpressed KCNQ10T1-induced effects, and depletion of SMAD5 reversed the miR-19a-3p inhibitor-induced effects on osteogenic medium-treated BMSCs.

**Conclusions:**

Upregulation of KCNQ10T1 promoted osteogenic differentiation of BMSCs through miR-19a-3p/SMAD5 axis in bone fracture.

## Introduction

Bone fracture is a common orthopedic disease that needs over 3 months to recover. Delayed fracture union and nonunion are common and worrisome complications in fracture treatment, placing a significant burden on individuals and society [1, 2]. Despite the increasingly comprehensive exploration of fracture healing mechanisms, these influencing factors remain a major clinical challenge for fracture treatment [3]. Mesenchymal stem cells (MSCs), which are pluripotent stromal cells, have attracted much attention as powerful tools for tissue regeneration [4]. MSCs have the ability to self-renew and differentiate into multiple lineages, such as osteoblasts (bone), chondrocytes (cartilage), muscle cells (muscle), and fat cells (adipocytes) [5]. In addition, bone MSCs (BMSCs) are widely available from bone marrow, adipose tissue, cord blood, and any other tissue [6]. In particular, BMSCs tend to promote osteoblasts and stimulate bone formation. There is much convincing evidence that MSCs can repair bone and related defects in animal models [7, 8]. Systemic and local administration of allogeneic BMSCs promotes fracture healing in rats [9]. However, the ability of BMSCs to differentiate into functional osteoblasts remains limited in terms of bone regeneration in vivo. Therefore, stimulating osteogenic differentiation of BMSCs may be considered as a potential therapeutic approach to promote bone regeneration.

Long non-coding RNAs (lncRNAs) are endogenous cellular ribonucleic acid RNAs with a length of 200 nt ~ 100 kb [10]. Recently, several lncRNAs have been found to play an important role in the pathophysiological processes of various orthopedic diseases, such as LncRNA ROR [11], LncRNA THUMPD3-AS1 [12], lncRNA-CRNDE [13], etc. LncRNA-KCNQ1OT1 (KCNQ1OT1) is located on human chromosome 11p and is a chromatin regulatory RNA [14]. KCNQ1OT1 is a well-studied lncRNA, which has a profound impact on the regulation of colon cancer [15], non-small cell lung cancer [16], ischemic stroke [17] and osteogenic differentiation [18]. In the field of orthopedics, KCNQ1OT1 has been shown to accelerate osteoblast differentiation through up-regulating the Wnt/β-catenin signaling pathway [18]. In addition, KCNQ1OT1 silencing inhibited osteogenic differentiation and downregulated expression of osteogenic differentiation related proteins [19, 20]. However, the relationship between KCNQ1OT1 and growth or osteogenic differentiation of BMSCs is still not well defined.

MicroRNAs (miRNAs) are short non-coding RNAs, which are common in the expression of post transcriptional regulatory genes and mainly bind to the 3 '- untranslated region of targeted messenger RNA to regulate cell biological processes, including BMSCs differentiation [21–25]. miR-19a-3p is confirmed to be broadly conserved among vertebrates [26] and participate in the pathogenesis of preeclampsia and atherosclerosis [27, 28]. Recent studies have demonstrated the involvement of miR-19a-3p in the progression of various cancers including glioma, lung cancer, breast cancer, osteosarcoma, gastric cancer and hepatocellular carcinoma [29–32]. However, the precise mechanism by which miR-19a-3p in bone fracture treatment remains unknown. Here, through the Starbase and TargetScan on line database, we found that KCNQ1OT1 targeted miR-19-3p and Smad5 was a target gene of miR-19-3p. Smad5 is a receptor regulated SMAD protein that is a key transcription factor for osteogenic differentiation. Under physiological conditions, Smad5 is mainly located in the cytoplasm. When Smad5 is phosphorylated, it is directed to the nucleus, thereby regulating the expression of osteogenic genes and inducing osteogenic differentiation [33]. Inhibiting nuclear translocation of Smad5 can inhibit osteogenic differentiation of BMSCs [34].

Therefore, our study tended to investigate the molecular mechanisms of KCNQ1OT1 in bone fracture in vitro. We hypothesized that KCNQ1OT1 sponges miR-19-3p to regulate Smad5 expression to regulate osteogenic differentiation of BMSCs. Our research provided a novel understanding of bone fracture treatment.

## Materials and methods

### Cell culture

The mouse Bone Mesenchymal Stem Cells (BMSCs) were purchased from Beijing Baiou Bowei Biotechnology Co., Ltd (Beijing, China). The cells were cultured in complete α-MEM, and 10% fetal bovine serum and 1% penicillin streptomycin were added to the culture medium. The cultivation environment is set to 95% air and 5% CO2, and the temperature is set to 37 °C.

### Cell transfection

Overexpressed KCNQ10T1 vector (pcDNA3.1-KCNQ1OT1), overexpressed miR-19a-3p (miR-19a-3p mimic), suppressed miR-19a-3p (miR-19a-3p inhibitor), downregulated Smad5 (si-Smad5), as well as their negative control plasmids (pcDNA3.1, NC mimic, NC inhibitor, si-NC) in this study were all bought from GenePharma (Shanghai, China). BMSCs were cultured at a concentration of 2 × 10^5^ cells per well in 6-well plates. After the cells had grown to about 60% confluence, corresponding vectors were transfected into BMSCs using Lipofectamine 3000 reagent (Life Technologies,CA, USA), with all procedures following the manufacturer's protocol.

### Osteogenic differentiation

BMSCs were inoculated into a 12-well plate at 5 × 10^5^ cells/well, and cultured in medium containing 5 mmol/L β-glycerophosphate sodium, 50μg/mL vitamin C, 100 mmol/L dexamethasone and 10% FBS. The supernatant was discarded after 7 days, then BMSCs were fixed with 4% paraformaldehyde for 15 min, and stained with alkaline phosphatase (ALP) and Alizarin Red according to the instructions of kits (Beyotime, Shanghai, China) for observation and semi-quantitative analysis of mineralized nodules.

### Cell counting kit (CCK)-8 assay

BMSCs were planted into 96-well plates, followed by incubating for 0, 24, 48, and 72 h. 10 μl CCK-8 (KeyGEN, Jiangsu, China) was added into each well and incubated for 4 h. The absorption was measured at 450 nm using a microplate reader (BioTek, VT, USA).

### Flow cytometry

BMSCs were digested by trypsin and washed by PBS, and the cell suspension concentration was adjusted to 1 × 10^4^ cells/mL. The cells were incubated and stained by Annexin V-FITC and propidium iodide in dark for 15 min. The apoptosis rate of each group was detected by flow cytometry (BD FACSCalibur, NJ, USA).

### RT-qPCR

BMSCs of each group were inoculated into 6-well plates. RNA was isolated with TRIzol (PrimeScript™ RT Kit, Takara, Japan) and the mRNA was reversed into complementary DNA (cDNA) for RT-qPCR. The RT-qPCR was carried out using TaKaRa Ex Taq® kit (Takara, Japan). GAPDH was used as a housekeeping gene for KCNQ10T1 as well as Smad5 while U6 was applied as an internal reference of miR-19a-3p, and the relative expression levels of target genes were calculated using 2^−ΔΔCt^ (ΔCt = Ct target gene − Ct reference gene, Ct value represents the number of cycles when the fluorescence signal in each reaction tube reaches the set threshold).

### Western blot

Osteogenic differentiation-related proteins including ALP, osteocalcin (OCN), osteoprotegerin (OPG) and osteopontin (OPN) were measured in BMSCs. Total protein extracted from BMSCs was determined with the BCA kit (Sigma-Aldrich, MO, USA), and then subjected to 10% SDS-PAGE gel electrophoresis, and transferred onto the PVDF membrane (Millipore, MA, USA). The membranes were blocked with 5% milk for 2 h and then incubated with primary antibodies including anti-ALP(1/1000, Abcam, MA, USA), anti-OCN (1/1500, Abcam), anti-OPG (1/800, Abcam), anti-OPN (1/1000, Abcam) and GADPH (1/3000, Abcam) for 12 h at 4 °C. Subsequently, immune complexes were incubated with horseradish peroxidase-labeled Immunoglobulin G (IgG; 1/2000, Abcam) for 1 h and visualized using a chemiluminescence kit (Beyotime, Shanghai, China). Finally, the brands were photographed by IS gel image analysis system and analyzed using the Image J.

### Verification of binding relationship between mRNA and miRNA

Dual luciferase reporter assay as well as RNA pull-down methods were carried out to confirm the prediction of bioinformatics concerning the interactions between miR-19a-3p and KCNQ1OT1 of Smad5. The wild-type (WT) KCNQ10OT1, mutant-type (MUT) KCNQ1OT1, WT Smad5 3′-UTR, MUT Smad5 3′-UTR were synthesized and cloned into pmirGLO luciferase vectors (Promega, Beijing, China) to determine whether miR-19a-3p directly targets KCNQ1OT1 and the Smad5 3′-UTR. The miR-19a-3p mimic negative control plasmids (nc mimic), miR-19a-3p mimic (mimic) along with Renilla luciferase plasmid (Promega, Beijing, China) were co-transfected into BMSCs. Firefly and Renilla luciferase activities were determined by a Dual-Luciferase Reporter Assay kit (BioVision Tech, Guangdong, China). As for RNA pull-down method, 500 μg streptavidin magnetic beads were combined with 200 pmol biotin-labeled miR-19a-3p mimic, and added into RNA extracted from BMSCs. Eluting buffer was added to collect the pulled RNA complex after 30 min incubation at room temperature, and the KCNQ1OT1 and Smad5 levels were quantitatively analyzed by RT-qPCR.

### Statistical analysis

For bioinformatic analysis, the target miRNA of KCNQ1OT1 was predicated by Starbase online database (http://starbase.sysu.edu.cn/), and the target gene of miR-19a-3p was predicated by TargetScan online database (http://www.targetscan.org/). The results were statistically analyzed using GraphPad Prism 9.0 (MacKiev Software). Normal distribution (Shapiro–Wilk) and homogeneity of variance test were carried out for multiple groups of data, and those who met the criteria were represented in the form of "$\overline{x} \pm {\text{sd}}$". One-way ANOVA and paired T test of two independent samples were used for comparison between groups and populations. Each experiment was conducted independently three times (*n* = 3). *p* < 0.05 on both sides was considered statistically significant.

## Results

### KCNQ1OT1 overexpression promoted the osteogenic differentiation of BMSCs

**Fig. 1:**
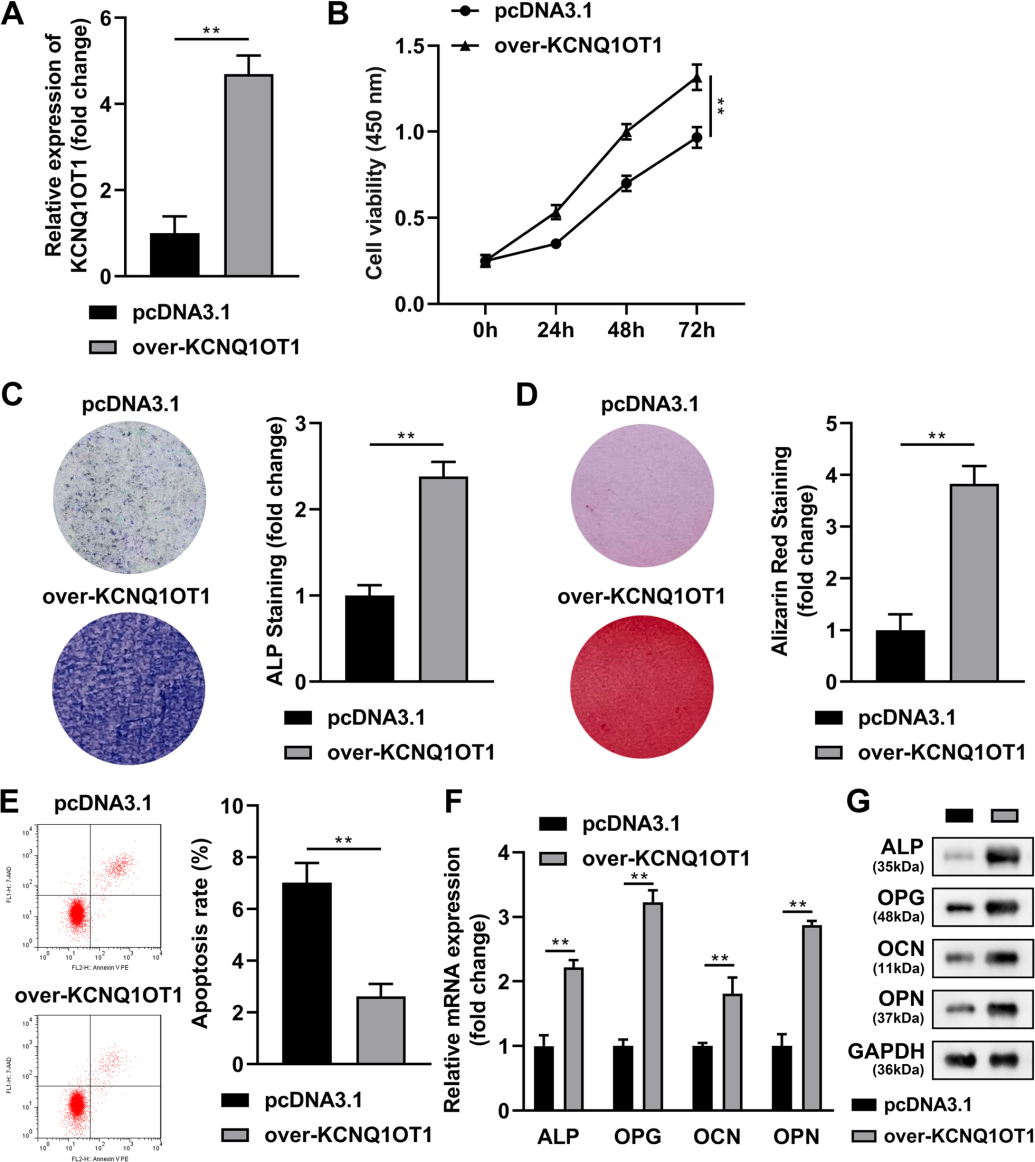
KCNQ1OT1 overexpression promoted the osteogenic differentiation of BMSCs. **A** Overexpression efficiency of pcDNA3.1-KCNQ1OT1 was detected by RT-qPCR assay. Then the BMSCs were cultured in osteogenic medium and transfected with pcDNA3.1-KCNQ1OT, **B** the cell viability was detected by CCK-8 assay. The positive mineralized nodules were stained by ALP (**C**) as well as Alizarin Red (**D**). **E** The apoptosis rate was measured by flow cytometry. The mRNA (**F**) and protein (**G**) expressions of ALP, OPG, OCN and OPN were detected by RT-qPCR and western blot assays. **P < 0.01

Firstly, we explored the role of KCNQ1OT1 in the osteogenic differentiation of BMSCs. After pcDNA3.1-KCNQ1OT1 (over-KCNQ1OT1) transfection, KCNQ1OT1 was obviously upregulated (Fig. 1A). KCNQ1OT1 overexpression significantly increased the cell viability of osteogenic medium-treated BMSCs (Fig. 1B). In addition, positive mineralized nodules stained by ALP (Fig. 1C) as well as Alizarin Red (Fig. 1D) in osteogenic medium-treated BMSCs transfected with over-KCNQ10T1 were markedly more than that in negative control group. Besides, KCNQ1OT1 overexpression significantly decreased the apoptosis rate of the osteogenic medium-treated BMSCs (Fig. 1E). Moreover, overexpression of KCNQ1OT1 induced the significant upregulation of mRNA (Fig. 1F) and protein (Fig. 1G) expressions of ALP, OPG, OCN and OPN in osteogenic medium-treated BMSCs.

### KCNQ1OT1 targets miR-19a-3p

**Fig. 2:**
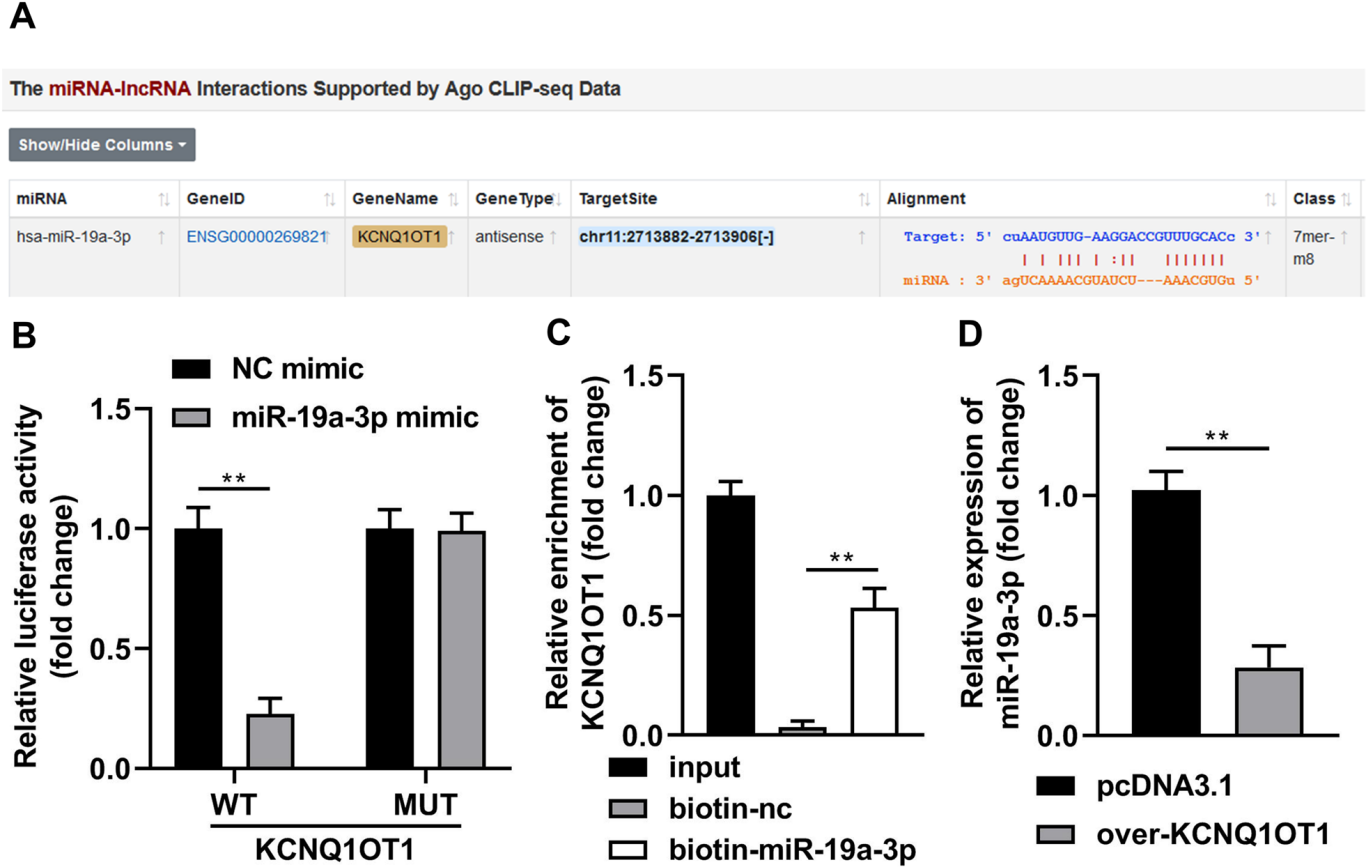
KCNQ1OT1 targets miR-19a-3p. **A** The binding site between KCNQ1OT1 and miR-19a-3p was predicted by Starbase online database. The binding interaction between KCNQ1OT1 and miR-19-3p was tested by decreased luciferase reporter (**B**) and RNA pull-down (**C**) assays. **D** The miR-19a-3p levels were detected by RT-qPCR after KCNQ1OT1 overexpression. **P < 0.01

The results of bioinformatic analysis showed that KCNQ1OT1 could binds to miR-19a-3p (Fig. 2A). MiR-19a-3p mimic rather than nc mimic dramatically decreased luciferase activity after co-transfected with WT-KCNQ1OT1; whereas, miR-19a-3p co-transfected with MUT-KCNQ1OT1 did not affect luciferase activity (Fig. 2B). Results of RNA pull-down method revealed that KCNQ1OT1 was obviously captured by biotin-miR-19a-3p, compared with biotin-nc (Fig. 2C). In addition, after KCNQ1OT1 overexpression, the miR-19a-3p levels were significantly decreased in the BMSCs (Fig. 2D).

### Elevated KCNQ10T1 accelerates osteogenic differentiation of BMSCs via sponging miR-19a-3p

**Fig. 3:**
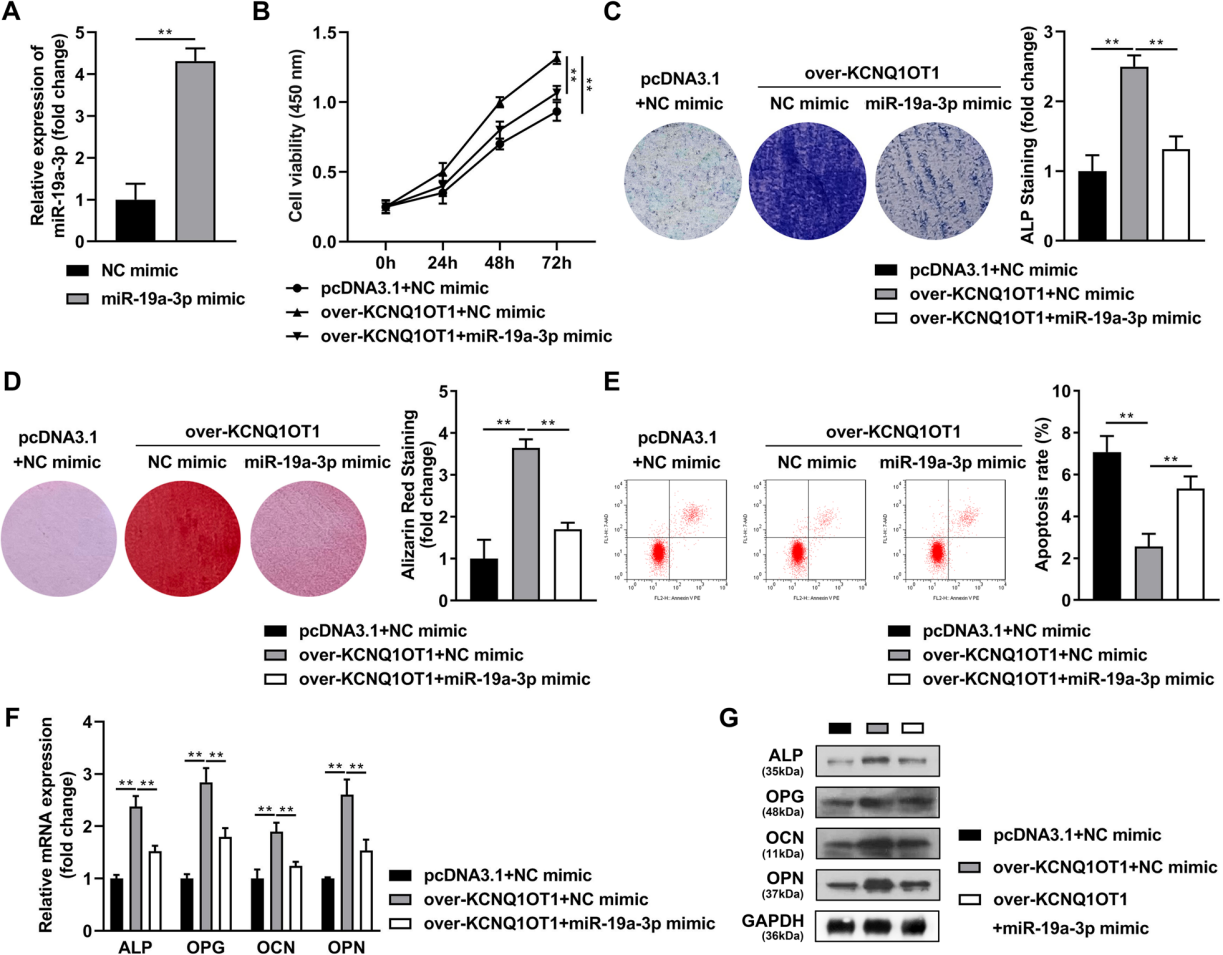
Elevated KCNQ10T1 accelerates osteogenic differentiation of BMSCs via sponging miR-19a-3p. **A** Overexpression efficiency of miR-19a-3p mimic was detected by RT-qPCR assay. Then the BMSCs were cultured in osteogenic medium and transfected with pcDNA3.1-KCNQ1OT and miR-19a-3p mimic, **B** the cell viability was detected by CCK-8 assay. The positive mineralized nodules were stained by ALP (**C**) as well as Alizarin Red (**D**). **E** The apoptosis rate was measured by flow cytometry. The mRNA (**F**) and protein (**G**) expressions of ALP, OPG, OCN and OPN were detected by RT-qPCR and western blot assays. **P < 0.01

After successfully transfection, miR-19a-3p in BMSCs was notably increased in the mimic group (Fig. 3A). Increased cell viability (Fig. 3B), positive mineralized nodules intensity stained by ALP (Fig. 3C) and Alizarin Red (Fig. 3D), and decreased apoptosis were induced by overexpressed KCNQ1OT1 (Fig. 3E), which were obviously reversed by overexpressed miR-19a-3p (Fig. 3B–E). Likewise, the increase in mRNA (Fig. 3F) and protein (Fig. 3G) levels of ALP, OPG, OCN and OPN induced by overexpressed KCNQ1OT1 was significantly decreased after miR-19a-3p overexpression.

### miR-19a-3p directly targets Smad5

**Fig. 4:**
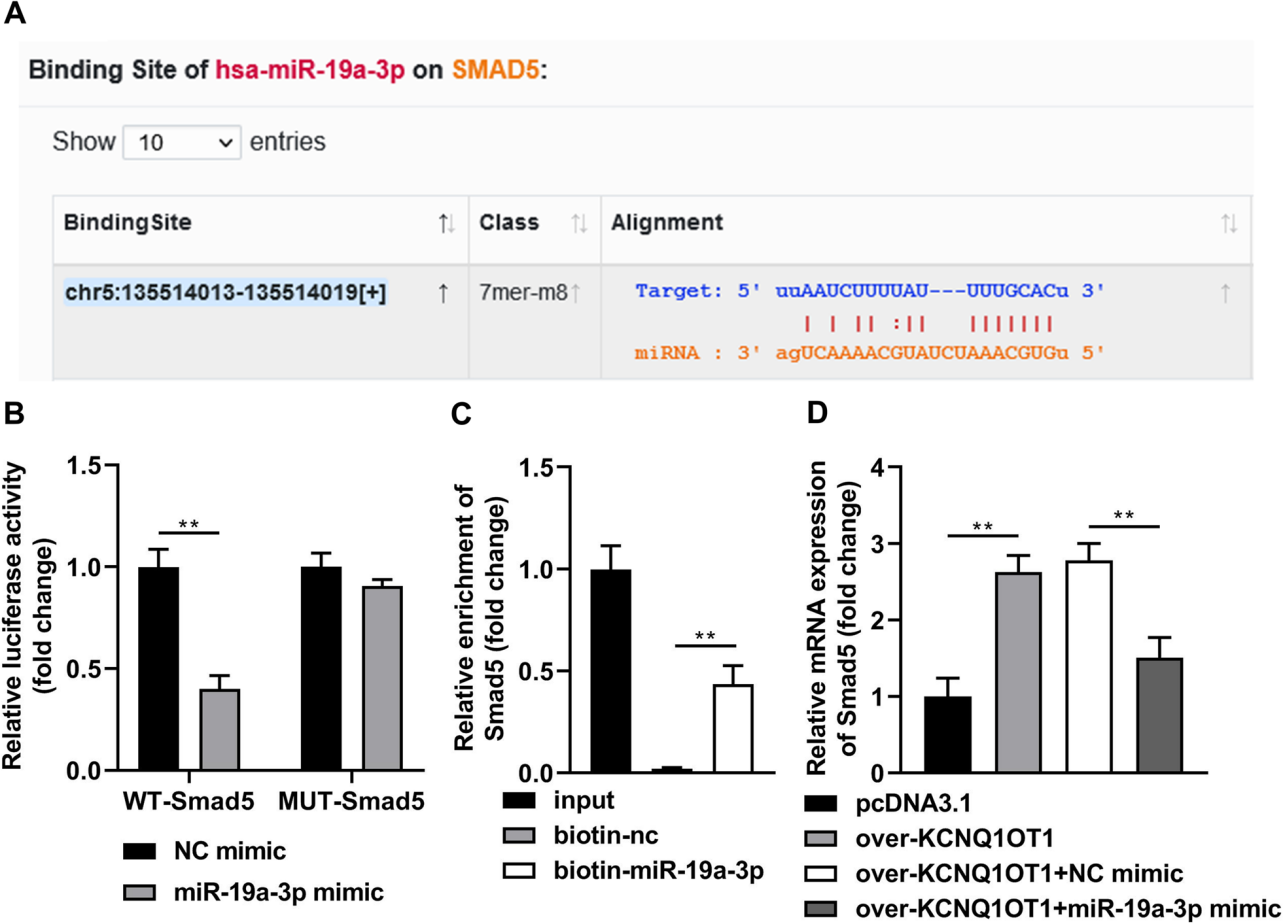
miR-19a-3p directly targets Smad5. **A** The binding site between Smad5 and miR-19a-3p was predicted by TargetScan online database. The binding interaction between Smad5 and miR-19-3p was tested by decreased luciferase reporter (**B**) and RNA pull-down (**C**) assays. **D** The Smad5 levels were detected by RT-qPCR after KCNQ1OT1 and miR-19a-3p overexpression. **P < 0.01

The results of bioinformatics indicated that Smad5 has the binding sites in miR-19a-3p (Fig. 4A). MiR-19a-3p mimic rather than nc mimic dramatically decreased luciferase activity after co-transfected with WT-Smad5; whereas, miR-19a-3p co-transfected with MUT-Smad5 did not affect luciferase activity (Fig. 4B). Results of RNA pull-down method revealed that Smad5 was obviously captured by biotin-miR-19a-3p, compared with biotin-nc (Fig. 4C). The expression of Smad5 in the osteogenic medium-treated BMSCs was dramatically increased after KCNQ1OT1 overexpression, which was decreased after miR-19a-3p overexpression (Fig. 4D).

### Silenced Smad5 inhibited the role of downregulated miR-19-3p in BMSCs

**Fig. 5:**
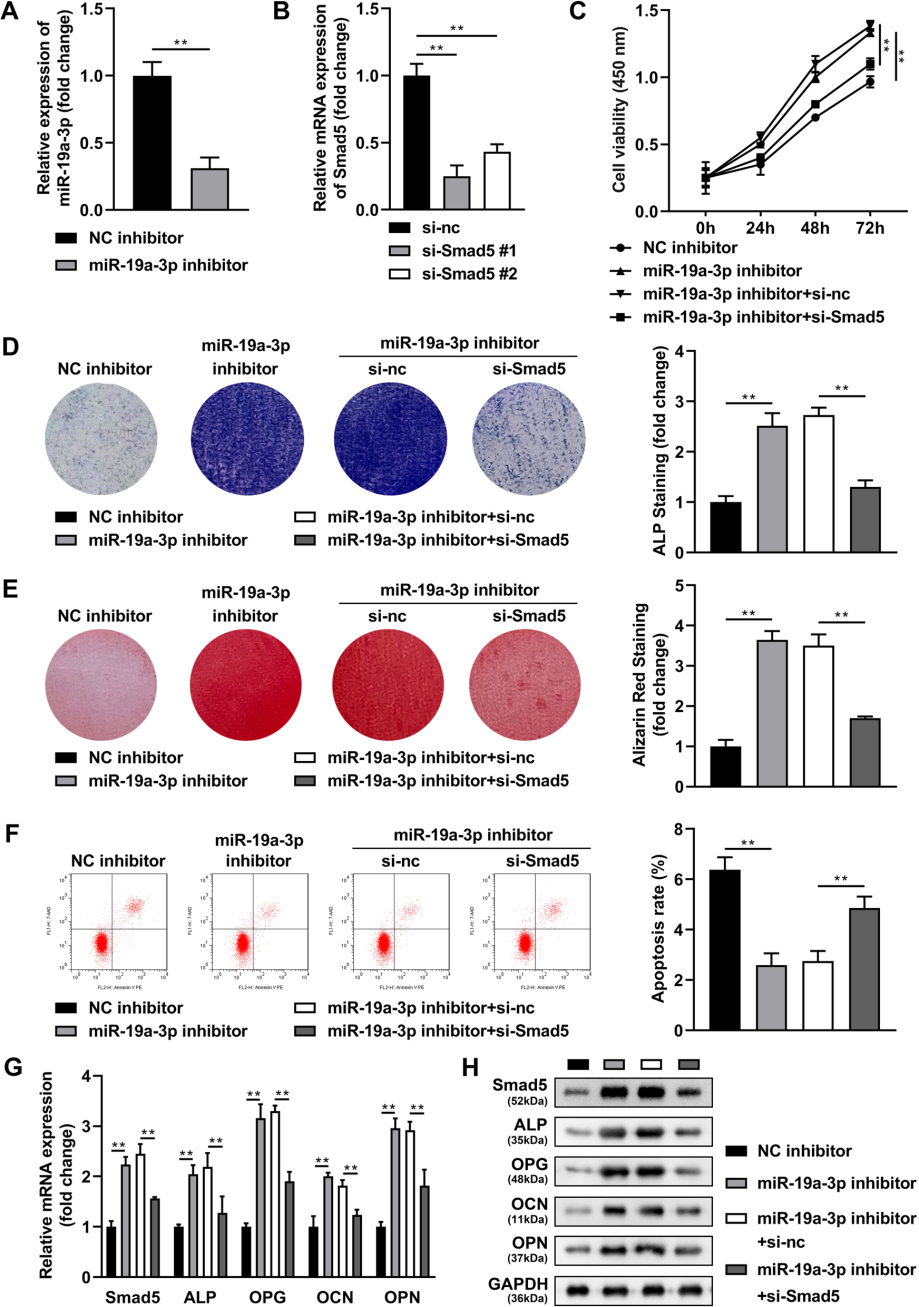
Silenced Smad5 inhibited the role of downregulated miR-19-3p in BMSCs. Knockout efficiency of miR-19a-3p inhibitor (**A**) and si-Smad5 (**B**) was detected by RT-qPCR assay. Then the BMSCs were cultured in osteogenic medium and transfected with miR-19a-3p inhibitor and si-Smad5, **C** the cell viability was detected by CCK-8 assay. The positive mineralized nodules were stained by ALP (**D**) as well as Alizarin Red (**E**). **F** The apoptosis rate was measured by flow cytometry. The mRNA (**G**) and protein (**H**) expressions of Smad5, ALP, OPG, OCN and OPN were detected by RT-qPCR and western blot assays. **P < 0.01

The knockout efficiency of miR-19a-3p inhibitor and si-Smad5 was test by PCR assay. MiR-19a-3p inhibitor significantly decreased the miR-19a-3p levels (Fig. 5A) and si-Smad5 significantly decreased the Smad5 levels (Fig. 5B). Increased cell viability (Fig. 5C), positive mineralized nodules intensity stained by ALP (Fig. 5D) and Alizarin Red (Fig. 5E), and decreased apoptosis were induced by miR-19a-3p inhibitor (Fig. 5F), which were obviously reversed by Smad5 knockdown (Fig. 5C–F). In addition, the increase in mRNA (Fig. 5G) and protein (Fig. 5H) levels of Smad5, ALP, OPG, OCN and OPN induced by miR-19a-3p inhibitor was significantly decreased after Smad5 knockdown.

## Discussion

In current study, we clarified the role of KCNQ1OT1 in bone fracture and the molecular mechanism in vitro. Heightening of KCNQ1OT1 helped to protect BMSCs from dysfunction by promoting osteogenic differentiation. Furthermore, KCNQ1OT1 played its role via the miR-19a-3p/Smad5 axis.

Accumulating evidences have showed that lncRNAs are regarded as the regulator of bone fracture occurrence and development [35]. The abnormally expressed lncRNAs played a different role in osteogenic differentiation of BMSCs. For example, Zhang et al. [36] demonstrated that lncRNA-NEAT1 upregulated the expression of osteogenic differentiation proteins to improve mitochondrial function. It indicated that lncRNA-NEAT1 might be a potential therapeutic target for skeletal aging. Yin et al. [37] suggested that lncRNA-Malat1 knockdown suppressed the osteogenic differentiation of BMSCs, which was reversed by decreasing the expression of miR-129-5p. In the pathogenesis of osteoporosis, high levels of lncRNA SNHG1 increased the expression of DNMT1 via interacting with PTBP1. LncRNA SNHG1 contributed to osteoporosis through leading to osteoprotegerin hypermethylation and downregulated osteoprotegerin expression [38]. Thus, it can be seen, focusing on the role of differentially expressed lncRNAs in osteogenic differentiation of BMSCs may be the key to treating orthopedic diseases such as fractures and osteoporosis. KCNQ1OT1, a widely studied lncRNA, has been shown to exhibit different expression levels in different diseases. For instance, in the osteosarcoma [39], ovarian cancer [40], lung squamous cell carcinoma [41], etc. High levels of KCNQ1OT1 promoted the malignant behaviors, such as excessive proliferation. In other diseases, such as atherosclerosis, high levels of KCNQ1OT1 prevented cholesterol efflux and induced lipid accumulation in THP-1 macrophages. In contrast, KCNQ1OT1 silencing protected against atherosclerosis in apoE-/-mice and inhibited the lipid accumulation in THP-1 macrophages [42]. However, in the process of cellular senescence, high levels of KCNQ1OT1 inhibited senescence-associated heterochromatin foci, transposon activation and retrotransposition as well as cellular senescence, suggesting KCNQ1OT1 inhibited the cellular senescence. Here, we found that KCNQ1OT1 overexpression promoted the growth and osteogenic differentiation of BMSCs. Our results were similar to a previous study, which also demonstrated KCNQ1OT1 promoted osteogenic differentiation of BMSCs through inhibiting miR-205-5p [43].

More and more evidence suggests that lncRNAs act as competitive endogenous RNAs (ceRNAs) to sponge miRNAs [44]. As reported by previous studies, KCNQ1OT1 has been demonstrated to sponge miR-34c-5p in osteosarcoma [39], miR-125b-5p in ovarian cancer [40], miR-26a-5p in ischemia reperfusion [45]. Here, through the Starbase online database, we found that KCNQ1OT1 targeted to miR-19a-3p. miR-19a-3p has been demonstrated to participated in various diseases such as myocardial ischemia/reperfusion injury [46], sepsis-induced lung injury [47], in multiple myeloma [48], etc. Most studies have found that miR-19a-3p acts as a sponge for lncRNAs, thereby participating in the progression of diseases. For example, Xiang et al. [49] found that miR-19a-3p promoted the migration and epithelial–mesenchymal transition of breast cancer cells through sponge adsorbing by LINC00094. In osteoporosis, Chen et al. [50] demonstrated that lncRNA Xist was a sponge of miR-19a-3p to inhibit BMSCs osteogenic differentiation. Similarly, this study found that miR-19a-3p overexpression inhibited the BMSCs osteogenic differentiation and reversed the role of KCNQ1OT1 in the BMSCs. However, there are contradictions with previous research, Chen et al. [50] exhibited the promoting effect of miR-19a-3p on osteogenic differentiation of BMSCs, while we confirmed the inhibitory effect of miR-19a-3p on osteogenic differentiation of BMSCs. We speculated that this may be due to they performed the study using BMSCs in aging cell models, while we are explored the osteogenic differentiation of normal BMSCs. In addition, different lncRNAs and target genes may lead to different expressions and functions of miR-19a-3p. Therefore, further research is still needed to explore the specific mechanism of miR-19a-3p in BMSCs.

Finally, we confirmed that Smad5 was a target gene of miR-19a-3p. Smad5 is a receptor regulated Smad protein that is a key transcription factor for osteogenic differentiation [34]. Under physiological conditions, Smad5 is mainly located in the cytoplasm. When Smad5 is phosphorylated, it is directed to the nucleus, thereby regulating the expression of osteogenic genes and inducing osteogenic differentiation [51]. According to reports, inhibiting nuclear translocation of p-Smad5 can inhibit osteogenic differentiation of BMSCs [52]. Here, we found that Smad5 knockdown reversed the effects of miR-19a-3p on the growth and osteogenic differentiation of BMSCs. The findings suggested that miR-19a-3p targets Smad5 to promote bone fracture development. Taken together, Upregulation of KCNQ10T1 attenuated bone fracture progression by the miR-19-3p/Smad5 axis.

In conclusion, our research suggested KCNQ10T1 overexpression promoted the osteogenic differentiation of BMSCs. MiR-19a-3p overexpression reversed the role of KCNQ10T1 accelerates osteogenic differentiation of BMSCs via sponging miR-19a-3p. In addition, silenced Smad5 inhibited the role of downregulated miR-19-3p in BMSCs. KCNQ10T1 acted as a ceRNA to regulate osteogenic differentiation of BMSCs via miR-19-3p/Smad5 axis. Overexpression of KCNQ10T1 may be an alternative for the treatment of bone fracture. However, there was still a limitation in this study. Due to limitations in hospital research conditions, we did not conduct animal experiments to verify the role of KCNQ10T1 in bone growth and development in vivo. In the future, we will aim to establish a fracture mouse model and inject KCNQ10T1 overexpression lentivirus to further investigate the role of KCNQ10T1 in vivo.

## Data Availability

The datasets used and/or analyzed during the current study are available from the corresponding author on reasonable request.
